# Femoral head decentration on hip MRI: comparison between imaging planes, methods of contrast administration, and hip deformities

**DOI:** 10.1186/s13244-024-01777-7

**Published:** 2024-08-01

**Authors:** Florian Schmaranzer, Tadeus A. Becker, Alexander F. Heimann, Jose Roshardt, Joseph M. Schwab, Stephen B. Murphy, Simon D. Steppacher, Moritz Tannast, Till D. Lerch

**Affiliations:** 1 Department of Diagnostic-, Interventional-, and Pediatric Radiology, Inselspital, Bern University Hospital, University of Bern, Freiburgstrasse 20, 3010 Bern, Switzerland; 2 Department of Radiology, Balgrist University Hospital, Forchstrasse 340, 8008 Zurich, Switzerland; 3 Department of Orthopaedic Surgery, Inselspital Bern, University Hospital, University of Bern, Freiburgstrasse 18, 3010 Bern, Switzerland; 4 Department of Orthopaedic Surgery, HFR—Cantonal Hospital, University of Fribourg, Chemin des Pensionnats 2–6, 1700 Fribourg, Switzerland; 5 Center for Computer Assisted & Reconstructive Surgery, New England Baptist Hospital, 125 Parker Hill Avenue, Boston, MA 02120 USA

**Keywords:** Hip MRI, Hip instability, Hip arthroscopy, Femoroacetabular impingement, Hip dysplasia

## Abstract

**Objectives:**

To compare the prevalence of femoral head decentration (FHD) on different MR imaging planes in patients undergoing direct/indirect hip MR arthrography (MRA) with asymptomatic controls and to evaluate its association with osseous deformities.

**Methods:**

IRB-approved retrospective single-center study of symptomatic hips undergoing direct or indirect hip MRA at 3 T. Asymptomatic participants underwent non-contrast hip MRI at 3 T. FHD was defined as a continuous fluid layer between the acetabulum and femoral head and assessed on axial, sagittal and radial images. The association of intra-articular/intra-venous contrast agents and the prevalence of FHD was evaluated. The association of FHD with osseous deformities and joint damage was assessed using multiple logistic regression analysis.

**Results:**

Three-hundred ninety-four patients (447 hips, mean age 31 ± 9 years, 247 females) were included and compared to 43 asymptomatic controls (43 hips, mean age 31 ± 6 years, 26 females). FHD was most prevalent on radial images and more frequent in symptomatic hips (30% versus 2%, *p* < 0.001). FHD prevalence was not associated with the presence/absence of intra-articular contrast agents (30% versus 22%, OR = 1.5 (95% CI 0.9–2.5), *p* = 0.125). FHD was associated with hip dysplasia (OR = 6.1 (3.3–11.1), *p* < 0.001), excessive femoral torsion (OR = 3.0 (1.3–6.8), *p* = 0.010), and severe cartilage damage (OR = 3.6 (2.0–6.7), *p* < 0.001).

**Conclusion:**

While rare in asymptomatic patients, femoral head decentration in symptomatic patients is associated with osseous deformities predisposing to hip instability, as well as with extensive cartilage damage.

**Critical relevance statement:**

Decentration of the femoral head on radial MRA may be interpreted as a sign of hip instability in symptomatic hips without extensive cartilage defects. Its presence could unmask hip instability and yield promise in surgical decision-making.

**Key Points:**

- The best method of identifying femoral head decentration is radial MRI.
- The presence/absence of intra-articular contrast is not associated with femoral head decentration.
- Femoral head decentration is associated with hip deformities predisposing to hip instability.

**Graphical Abstract:**

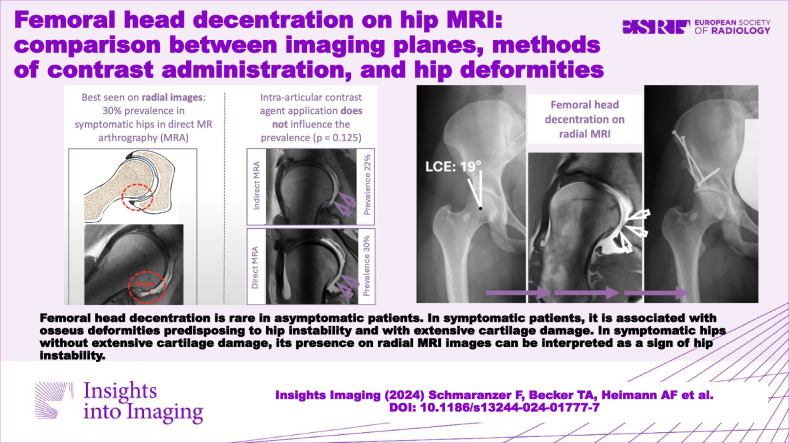

## Introduction

Diagnosis of hip instability can be challenging in patients eligible for joint preservation surgery, as the pathologies are often subtle and present with concomitant femoroacetabular impingement (FAI) [1]. However, it is important to identify these patients, as unstable hips are typically corrected with osteotomies [2], while isolated FAI focuses on resection of the osseous deformity. Conventional radiographic signs of obvious joint instability include a markedly reduced lateral center-edge (LCE) angle [3], an interrupted Shenton line [4], or an apparent joint space narrowing [5]. Attempts have been made to identify additional parameters to detect more subtle forms of hip instability. In this context, slight decentration of the femoral head with subsequent gadolinium collection between the posterior femur and acetabulum on magnetic resonance arthrography (MRA) has been proposed as a potential sign of hip joint instability [6, 7]. However, it is not yet clarified on which MR imaging plane this new potential sign of hip instability is best seen and whether the joint distension caused by intra-articular contrast injection affects its presence. To date, only symptomatic patients have been studied so far and the prevalence of femoral head decentration (FHD) in asymptomatic volunteers is unknown. In addition, its association with a broad range of hip deformities or with severe joint degeneration is unclear.

Therefore, our aim was to compare the prevalence of FHD on different MR imaging planes in patients undergoing direct or indirect MRA of the hip with an asymptomatic control group and to assess the association between FHD and different osseous deformities and patient demographics.

## Materials and methods

### Patients

**Fig. 1:**
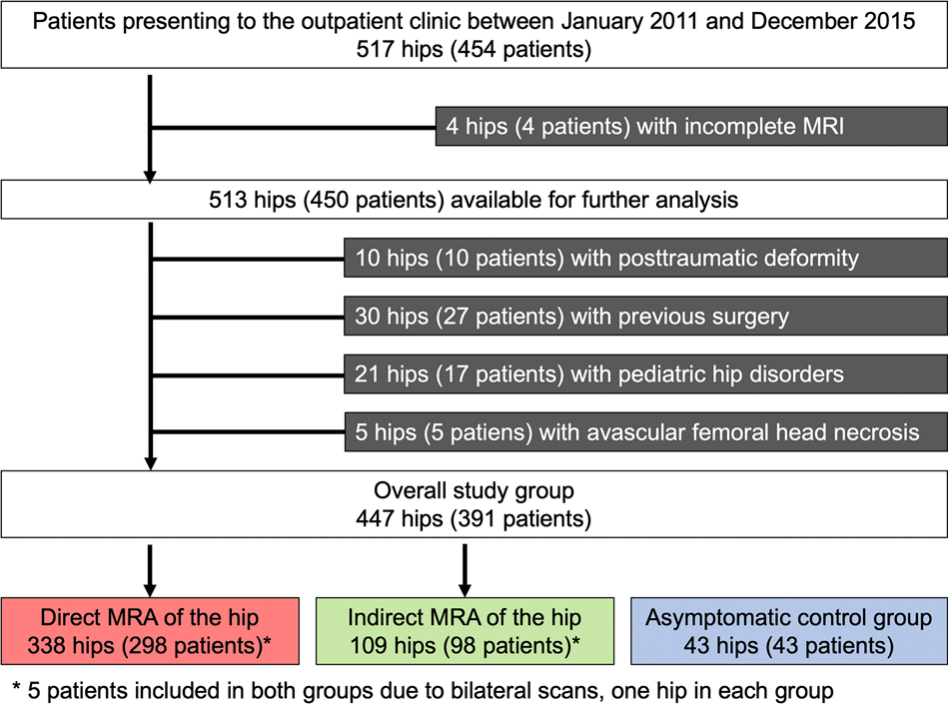
Flow chart with inclusion and exclusion criteria

Following institutional review board approval, we conducted a retrospective diagnostic study on patients with hip pain who presented to our tertiary center for joint-preserving hip surgery between January 2011 and December 2015. The inclusion criterion was biplanar radiographs and hip MRI according to the institutional protocol including images of the distal femoral condyles for measurement of femoral torsion. During the period in question, hip MRI was performed either as direct MRA or alternatively as indirect MR arthrography for subsequent post-contrast T1 mapping [8]. Applying these criteria to our institutional database yielded 517 hips (454 patients) available for further analysis. Following exclusion, the cohort was divided according to the MRA technique used: direct MRA and indirect MRA. In addition, 43 hips of 43 asymptomatic participants prospectively underwent non-contrast MRI of the hip and served as the control group (Fig. 1). Inclusion criteria were no history of hip pain and absence of hip pain on a clinical examination performed by two orthopedic residents (T.A.B. and J.R.).

### Diagnostic imaging

All symptomatic hips underwent conventional radiographic imaging with anteroposterior pelvis radiographs and axial cross-table views in the supine position. All MRAs were performed at 3 T (Prisma; Siemens Healthcare) using a large, flexible body coil. For direct MRA, 12–16 mL were injected into the hip joint under fluoroscopic guidance comprising 1–2 mL iodinated contrast agent (iopamidol, 200 mg/mL; Iopamiro 200; Bracco), 1–2 mL local anesthetic (ropivacaine hydrochloride; 2 mg/mL; Fresenius), and 10–12 mL diluted MRI contrast agent (gadopentetate-dimeglumine, 2.0 mmol/L; Magnevist; Bayer Healthcare). Indirect MRA was performed with intravenous administration of MRI contrast agent (gadopentetate-dimeglumine, 0.2 mmol/mL/kg, Magnevist, Bayer Healthcare) and a delay of 40 minutes between injection and image acquisition. The sequence protocol for direct- and indirect MRA included: coronal-, sagittal- and radial proton density-weighted turbo spin echo sequences of the hip without fat saturation. For measurement of femoral torsion T1-weighted turbo spin echo sequences of the hip and of the knee were acquired directly one after another. For ethical reasons, no radiographs were taken in the asymptomatic control group and a non-contrast MRI of the hip was performed with an axial-oblique 3D T2-weighted double echo steady state sequence which allowed for reformation of axial, sagittal, and radial images. The sequencing protocol is summarized in Supplementary Table 1.

### Assessment of femoral head decentration

**Fig. 2:**
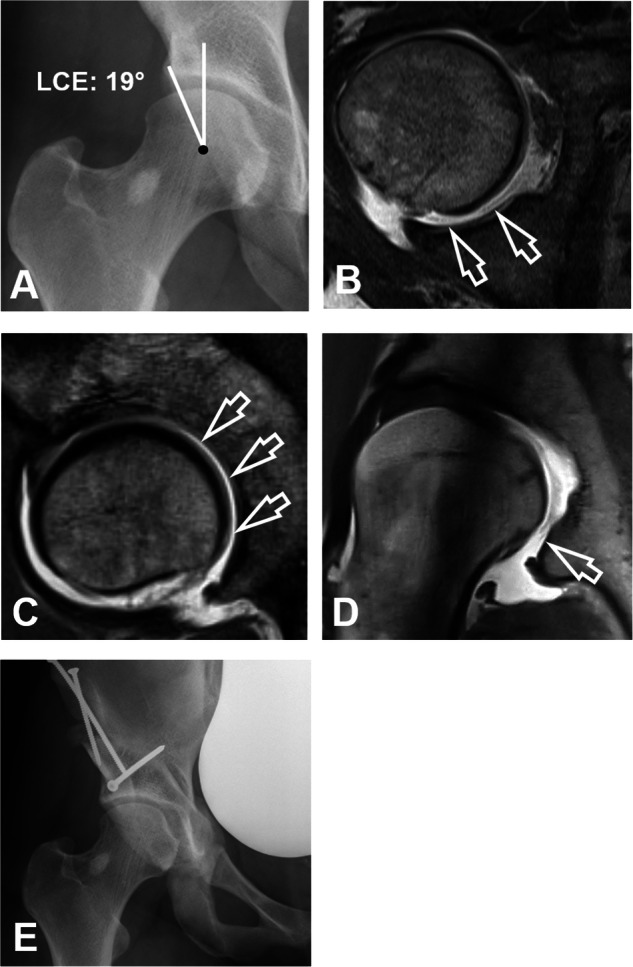
23-year-old woman with hip pain. **A** AP pelvis radiograph showing acetabular dysplasia with a decreased lateral center-edge (LCE) angle of 19°. The patient was referred to direct MR arthrography of the hip. **B** Axial-, (**C**) sagittal- and (**D**) radial proton density-weighted turbo spin echo images showing contrast interposition (arrows) between the posterior femur and the acetabulum consistent with femoral head decentration. **E** Postoperative AP pelvis radiograph after periacetabular osteotomy for correction of deficient acetabular coverage and hip instability

**Fig. 3:**
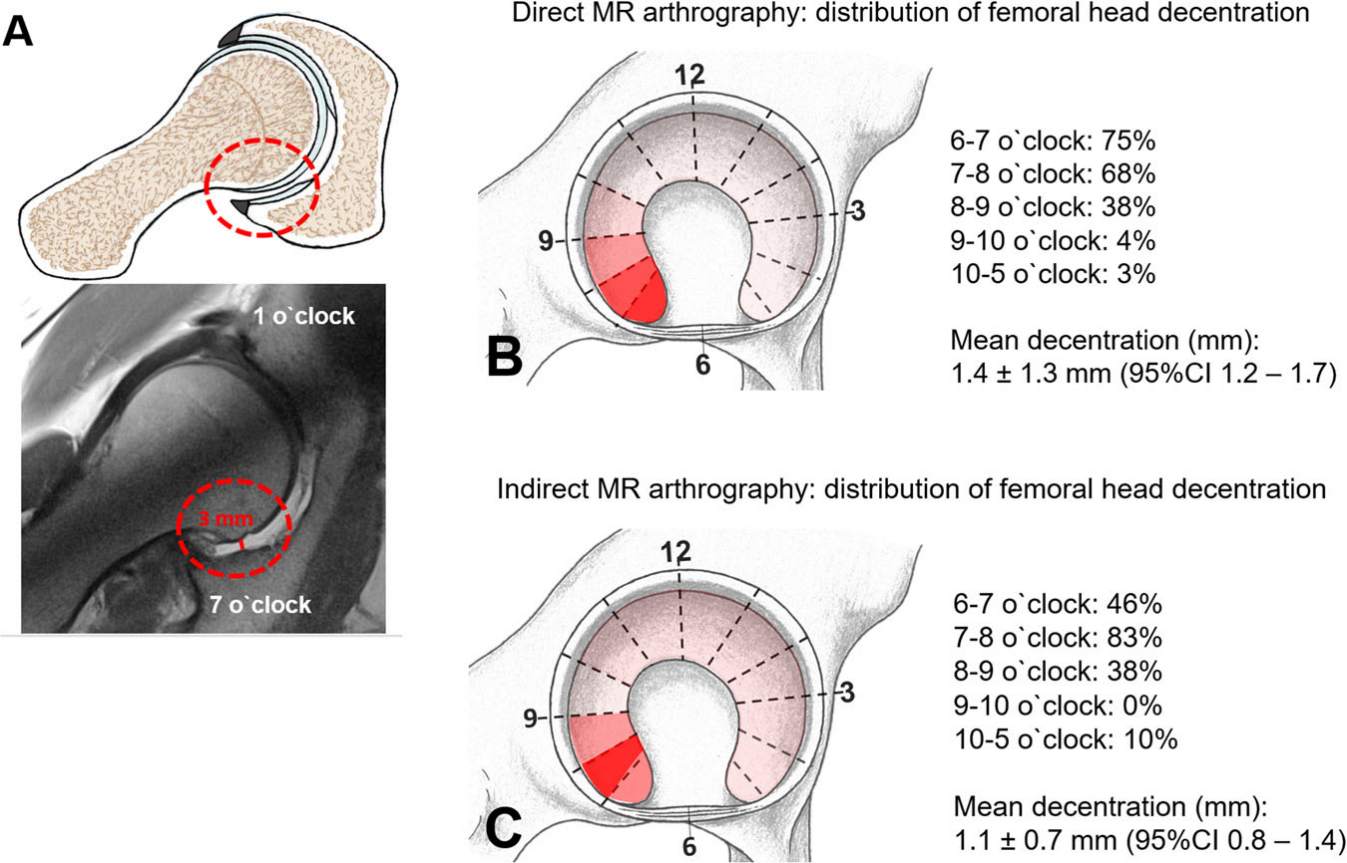
**A** Schematic drawing and corresponding radial proton density turbo spin echo image from indirect MR arthrography. Assessment of maximum decentration distance at the 7 o’clock position is shown. The width of the fluid layer between the femoral head and the acetabulum is measured (red circle with red line). No fluid layer is seen on the opposed antero-superior acetabulum at the 1 o’clock position. Topographical distribution of femoral head decentration around the clock-face on (**B**) direct- and (**C**) indirect MR arthrography of the hip. **B**, **C** Femoral head decentration was most frequently observed in the postero-inferior quadrant with comparable mean decentration distance (1.4 ± 1.3 mm versus 1.1 ± 0.7 mm; *p* = 0.194)

**Fig. 4:**
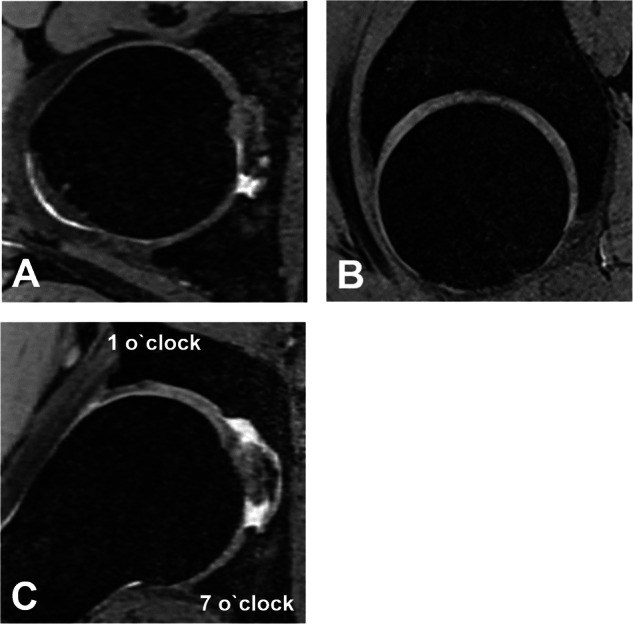
25-year-old female from the asymptomatic control group undergoing non-contrast hip MRI including a 3D T2-weighted double echo steady state sequence for reformation of (**A**) axial-, (**B**) sagittal-, and (**C**) radial images. **A**–**C** No continuous interposition of joint fluid between the acetabulum and femur consistent with femoral head decentration is detectable

Hip MRA and MRI were assessed for the presence of FHD on the axial, sagittal, and radial images by a radiologist (F.S.) with 7 years of experience in hip imaging. FHD was defined as a continuous layer of fluid signal visible between the femoral head and the acetabulum on at least one slice per imaging plane (Fig. 2). On radial images, the topographical distribution of FHD on the acetabular clock-face was assessed. Maximum decentration distance was measured perpendicular between the acetabulum and femur (Fig. 3). For the asymptomatic control group axial-, sagittal and radial images of the 3D T2-weighted double echo steady state sequence were reformatted for assessment of FHD (Fig. 4). Prevalence of FHD was compared between the three groups. In addition, a second radiologist (T.D.L.) assessed a subset (100 randomly selected hips in the direct and indirect MRA group and all 43 asymptomatic controls) for analysis of interobserver agreement for the diagnosis of FHD.

### Assessment of osseous deformities and joint degeneration

In the direct and indirect MRA groups, radiographic measurements were performed by an orthopedic resident (T.A.B.) with 4 years of experience and a radiologist with 7 years of experience in hip imaging (T.D.L.). Radiographic measurements of acetabular coverage (LCE) angle [9], acetabular index [10], extrusion index [3], anterior- and posterior femoral coverage [11]) assessment of retroversion signs (retroversion index [12], ischial spine sign [13], cross-over sign [12], and posterior wall sign [14]) and of the neck shaft angle [15] were performed with a previously validated software [16] (Hip2Norm, University of Bern). Alpha angles were measured on the cross-table views [17].

In addition, MRI measurement included central acetabular version and femoral torsion measured according to Murphy et al [18, 19]. All hips were graded for radiographic osteoarthritis according to Tönnis [20] and for severe cartilage damage on MRI. Severe cartilage damage on MRI was defined as damage extending over > 2 h on the clock-face or the presence of acetabular cysts. These findings were chosen since they reportedly are negative predictors for the outcome of joint-preserving hip surgery [21, 22]. Imaging parameters were compared between patients with and without FHD in both study groups.

### Association between osseous deformities, patient demographics, and joint degeneration with femoral head decentration

To determine the association between FHD and osseous deformities, six different pathomorphologies were analyzed: Hip dysplasia (LCE < 25°) [23], excessively high femoral torsion (> 35°) [24], valgus deformity (neck shaft angle > 140°) [15], cam deformity (alpha angle > 55°) [25], acetabular overcoverage (LCE > 35°) and acetabular retroversion (retroversion index > 30%, cross-over sign and ischial spine sign positive) [26]. In addition, advanced age > 40 years, female sex, Tönnis grade > 0, and severe cartilage damage on MRI [21, 22] were included in the analysis to account for potential confounding factors not related to osseous deformities. Single and multifactor analyses were performed to determine which factors are associated with FHD.

### Statistical analysis

Statistical analysis was performed using MedCalc (MedCalc Statistical Software, version 20.106, MedCalc Software Ltd, Ostend, Belgium). Normal distribution testing using a Kolmogorov-Smirnov test was carried out. We used the chi-square test to compare the prevalence of FHD both, between MR imaging planes, as well as in patients undergoing either direct and indirect MRA and the asymptomatic control group undergoing non-contrast hip MRI. The association between intra-articular/intra-venous contrast administration with FHD was assessed using single-factor regression analysis. To determine the interobserver reliability for the evaluation of the presence/absence of FHD Cohen’s kappa (*κ*) was used.

Depending on normal distribution testing, a comparison of the radiologic parameters between hips with/without FHD was performed using an independent samples t-test/Mann-Whitney test. A comparison of dichotomous parameters in hips with/without FHD was performed using chi-square tests. To investigate the relationship between FHD and the six osseous deformities including potential confounders, single-factor logistic regression analysis with calculation of the odds ratio (OR) and 95% confidence intervals (CI) was performed. Subsequently, stepwise multiple logistic regression analysis was performed for the retained factors.

## Results

### Patient characteristics

Of the 517 hips (454 patients), we excluded hips with incomplete MRI (4 hips), posttraumatic deformity (10 hips), previous surgery (30 hips), pediatric hip disorders (21 hips), or avascular necrosis of the femoral head (5 hips) (Fig. 1). The direct MRA group consisted of 338 hips of 296 patients (54% female) with a mean age of 31 ± 12 years. The indirect MRA group consisted of 109 hips of 98 patients (60% female) with a mean age of 31 ± 10 years. The control group consisted of 43 hips of 43 asymptomatic participants (60% female) with a mean age of 31 ± 6 years (Table 1).

**Table 1:** Demographic characteristics of the study groups

Parameter	Direct MRA (338 hips)	95% CI	Indirect MRA (109 hips)	95% CI	*p-*value	Control group (43 hips)
Age (y), mean ± SD	31 ± 12	30–32	31 ± 6	29–33	0.480	31 ± 6
Female sex	182 (54)	49–59	65 (60)	50–69	0.291	26 (60)
Right side	191 (57)	51–62	51 (47)	37–56	0.077	22 (51)
Bilateral	40 (12)	8–15	11 (10)	4–16	0.619	0 (0)
Osseous deformities						
Hip dysplasia (LCE < 25°)	93 (28)	23–32	21 (19)	12–27	0.086	n.a.
High femoral torsion (> 35°)	36 (11)	7–14	4 (4)	0–7	**0.027**	n.a.
Valgus deformity (neck shaft angle > 140°)	41 (12)	9–16	11 (10)	4–16	0.564	n.a.
Cam deformity (α angle > 55°)	177 (52)	46–57	86 (79)	70–86	**< 0.001**	n.a.
Acetabular overcoverage (LCE > 35°)	88 (26)	21–30	33 (30)	22–39	0.387	n.a.
Acetabular Retroversion^a^	71 (21)	17–25	29 (27)	18–35	0.223	n.a.

Values are depicted as *n* (%) if not otherwise noted

*MRA* magnetic resonance arthrography, *SD* standard deviation, *CI* confidence interval

Bold values indicate statistical significance *p* < 0.05

^a^ Defined as retroversion index of > 30%, cross-over, and ischial spine sign positive

### Femoral head decentration on different MR imaging planes

FHD showed the highest prevalence on radial images in all three study groups. In symptomatic hips undergoing direct MRA, the prevalence of FHD was highest on radial images (30%, 95% CI of 25–34%), followed by axial (12%, 9–16%) and sagittal (5%, 3–7%) images (*p* < 0.001). This was confirmed in the indirect MRA group in which FHD was most frequently detected on radial images (22%, 14–30%) (Table 2). On radial MRI mean decentration distance was comparable (*p* = 0.194) between direct and indirect MRA (1.4 ± 1.3 mm, 95% CI of 1.2–1.7 mm versus 1.1 ± 0.7 mm, 0.8–1.4 mm) and was detected in the majority of hips with FHD in the postero-inferior quadrant (06:00 to 09:00 o’clock) (Fig. 3).

**Table 2:** Frequency of femoral head decentration in different imaging planes

	MR imaging plane			*p*-value
Group	Sagittal	Axial	Radial	
Direct MRA group (338 hips)	17 (5)	41 (12)	100 (30)	**<** **0.001**
	3–7	9–16	25–34	
Indirect MRA group (109 hips)	2 (2)	5 (5)	24 (22)	**<** **0.001**
	0–4	1–9	14–30	
Control group (43 hips)	1 (2)	0 (0)	1 (2)	0.602
	0–7	0–0	0–7	

Values are depicted as *n* (%) and 95% confidence intervals

*MRA* magnetic resonance arthrography, *SD* standard deviation

Bold values indicate statistical significance *p* < 0.05

FHD on radial images was more frequent (both *p* < 0.001) in symptomatic hips, both in indirect- (22%, 95% CI of 14–30%) and direct MRA (30%, 25–34%), than in the asymptomatic control group (2%, 0–7%). In patients undergoing hip MRA, the overall prevalence of FHD on radial images was not associated (*p* = 0.125) with the method of contrast administration (direct versus indirect, OR = 1.5, 95% CI 0.9–2.5). Interrater reliability for the detection of FHD was almost perfect in all imaging planes among all three groups (Supplementary Table 2).

### Comparison between femoral head decentration and hip deformities in symptomatic patients

**Fig. 5:**
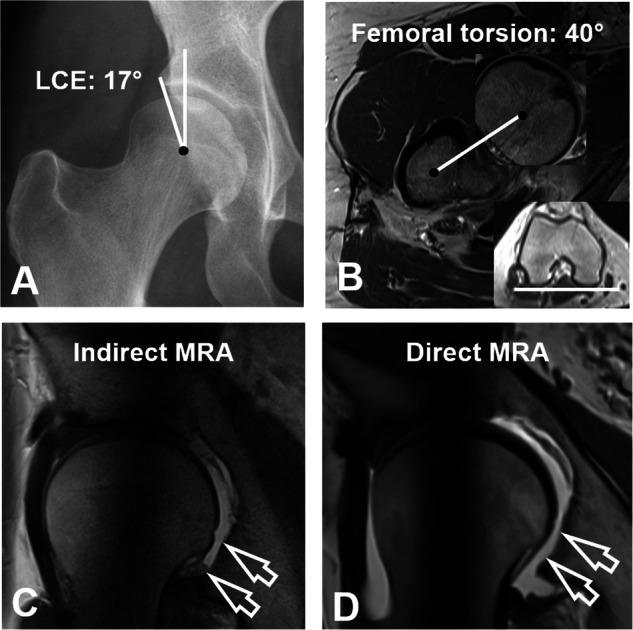
30-year-old woman with hip pain due to acetabular dysplasia and excessively high femoral torsion. **A** AP pelvis radiograph with a decreased lateral center-edge (LCE) angle of 17°. **B**, **C** The patient was referred to indirect MR arthrography of the hip. **B** Axial T1-weighted images show markedly increased femoral torsion of 40°. **C** Radial proton density-weighted turbo spin echo image shows postero-inferior femoral head decentration (arrows). **D** Two years later the patient underwent repeated imaging with direct hip MR arthrography with radial images showing femoral head decentration as well (arrows)

In general, hips with FHD had less lateral (lower LCE angle, higher extrusion index) and anterior femoral head coverage (less anterior femoral coverage), and a more shallow (higher acetabular index) and anteverted acetabulum (lower prevalence of retroversion signs) than hips without FHD (Table 3). Most importantly, hips with FHD had a lower LCE angle (both *p* < 0.001) in the direct (23 ± 9°, 95% CI of 22–25° versus 32 ± 7°, 31–33°) and indirect MRA group (25 ± 11°, 21–29° versus 33 ± 7°, 31–34°) (Table 3 and Fig. 2). Furthermore, hips with FHD exhibited higher (both *p* < 0.001) femoral torsion (24 ± 15°, 95% CI of 21–27° versus 16 ± 12°, 15–18°) and neck shaft angles (134 ± 7°, 133–136° versus 131 ± 6°, 130–132°) compared to hips without FHD (Fig. 5) in the direct MRA group.

**Table 3:** Comparison of imaging findings of patients with versus without femoral head decentration on radial images

Parameter	Direct MRA					Indirect MRA				
	Femoral head decentration on radial images									
	Present (100 hips)		Absent (238 hips)			Present (24 hips)		Absent (85 hips)		
	Mean ± SD	95% CI	Mean ± SD	95% CI	*p*	Mean ± SD	95% CI	Mean ± SD	95% CI	*p*
Lateral center-edge angle, °	23 ± 9	22–25	32 ± 7	31–33	**<** **0.001**	25 ± 11	21–29	33 ± 7	31–34	< **0.001**
Acetabular index, °	8 ± 8	7–10	2 ± 6	1–3	**<** **0.001**	7 ± 5	4–9	0 ± 5	−1 to 1	< **0.001**
Extrusion index, %	26 ± 9	24–28	18 ± 7	17–19	< **0.001**	26 ± 9	23–30	19 ± 6	18–21	< **0.001**
Anterior femoral coverage, %	21 ± 11	18–23	27 ± 8	26–28	< **0.001**	18 ± 9	15–22	26 ± 9	24–27	**0.001**
Posterior femoral coverage, %	41 ± 11	39–43	43 ± 9	42–44	0.141	43 ± 9	39–46	46 ± 9	44–48	0.082
Central acetabular version, °	21 ± 7	20–22	18 ± 7	17–18	< **0.001**	23 ± 6	21–25	16 ± 6	17–20	**0.002**
Retroversion index, *n* (%)	11 ± 14	8–14	11 ± 14	9–13	0.515	9 ± 12	4–14	14 ± 19	10–18	0.202
Ischial spine sign, *n* (%)	35 (35)	26–44	117 (49)	42–56	**0.017**	7 (29)	11–47	51 (60)	50–70	**0.008**
Cross-over sign, *n* (%)	78 (78)	70–86	211 (89)	85–93	**0.011**	11 (46)	26–66	58 (68)	58–78	**0.045**
Posterior wall sign, *n* (%)	84 (84)	77–91	203 (85)	81–90	0.762	19 (79)	63–96	53 (62)	52–73	0.126
Alpha angle, °	57 ± 11	55–59	57 ± 12	56–58	0.875	68 ± 11	64–73	63 ± 11	61–65	**0.042**
Neck shaft angle, °	134 ± 7	133–136	131 ± 6	130–132	< **0.001**	133 ± 8	129–136	131 ± 6	130–132	0.411
Femoral torsion, °	24 ± 15	21–27	16 ± 12	15–18	< **0.001**	21 ± 18	14–28	17 ± 9	15–19	0.527
Tönnis grade > 0, *n* (%)	24 (24)	16–32	61 (26)	20–31	0.753	3 (13)	0–25	12 (14)	7–22	0.840
Severe cartilage damage, *n* (%)	40 (40)	30–50	52 (22)	17–27	< **0.001**	8 (33)	15–52	9 (11)	4–17	**0.007**

Values are depicted as mean ± SD (95% CI) if not otherwise noted

*CI* confidence interval, *MRA* magnetic resonance arthrography, *SD* standard deviation

Bold values indicate statistical significance *p* < 0.05

**Fig. 6:**
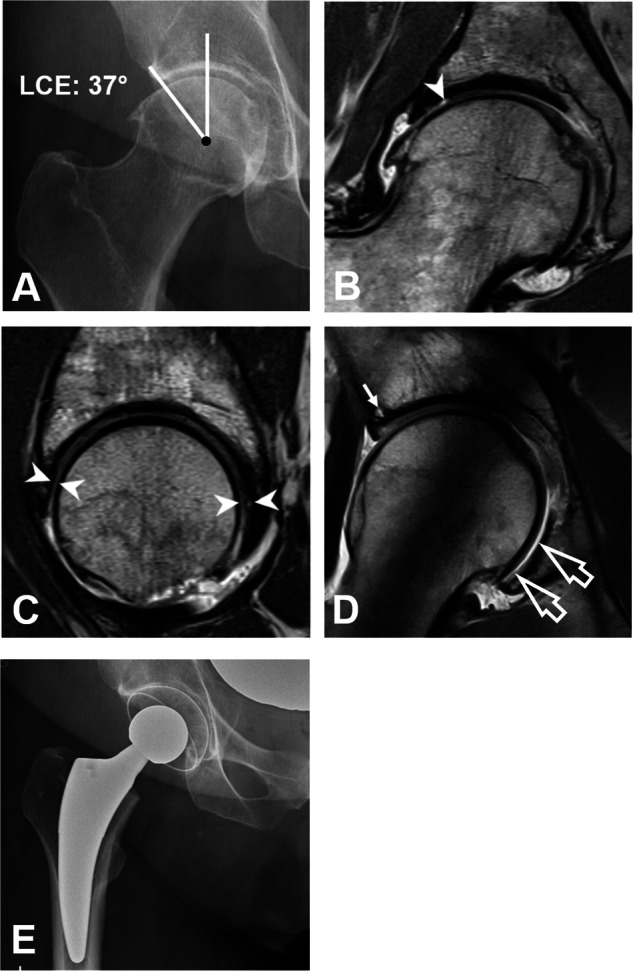
43-year-old man with hip pain due to mixed-type femoroacetabular impingement. **A** AP pelvis radiograph showing an increased lateral center-edge (LCE) angle of 37° and mild joint space narrowing. The patient underwent direct hip MR arthrography. **B** Coronal- and **C** sagittal- proton density-weighted turbo spin echo images showing cartilage damage involving more > 2 h on the clock-face (arrowheads). **D** Radial proton density-weighted turbo spin echo image demonstrates cartilage damage with a subchondral cyst (arrow). Femoral head decentration (open arrows) due to extensive joint damage is observed at the 7 o’clock position despite the presence of acetabular overcoverage. **E** Due to the advanced joint degeneration, the patient underwent subsequent total hip replacement

In both groups severe cartilage damage was more prevalent in hips with versus without FHD (Fig. 6). This was observed in the direct MRA (40%, 95% CI of 30–50% versus 22%, 17–27%; *p* < 0.001) as well as in the indirect MRA group (33%, 15–52% versus 11%, 4–17%; *p* = 0.007) (Table 3).

### Association between femoral head decentration and hip deformities in symptomatic patients

Single-factor and multiple, stepwise logistic regression analysis in the direct MRA group revealed higher odds for FHD with hip dysplasia (OR = 6.1, 95% CI of 3.3–11.1; *p* < 0.001), high femoral torsion (OR = 3.0, 1.3–6.8; *p* = 0.010), and valgus deformity (OR = 3.0, 1.4–6.7; *p* = 0.006). By contrast, the presence of acetabular overcoverage was protective against the presence of FHD (OR = 0.4, 0.2–0.9; *p* = 0.026) (Table 4). Similarly, the presence of hip dysplasia (OR = 5.2, 1.7–16.0; *p* = 0.004) and high femoral torsion (OR = 14.7, 1.2–175.7; *p* = 0.034) yielded higher odds for FHD in the indirect MRA group. In both groups, the direct (OR = 3.6, 2.0–6.7; *p* < 0.001) and indirect MRA group (OR = 4.3, 1.4–13.1; *p* = 0.009), we found severe cartilage damage to be associated with higher odds for FHD (Tables 4 and 5).

**Table 4:** Single-factor and multiple logistic regression analysis with odds ratios for the probability of femoral head decentration on radial images in direct MRA

Parameter	Single-factor logistic regression		Stepwise multiple logistic regression	
	OR (95% CI)	*p*-value	OR (95% CI)	*p*-value
Hip dysplasia	7.4 (4.4–12.7)	**<** **0.001**	6.1 (3.3–11.3)	**<** **0.001**
* LCE* < *25°*				
High femoral torsion	3.5 (1.7–7.0)	**<** **0.001**	3.0 (1.3–6.8)	**0.010**
* >* *35°*				
Valgus deformity	3.7 (1.9–7.1)	< **0.001**	3.0 (1.4–6.7)	**0.006**
* Neck shaft angle* > *140°*				
Cam deformity	0.8 (0.5–1.2)	0.261		
* Alpha angle* > *55°*				
Acetabular overcoverage	0.2 (0.1–0.4)	< **0.001**	0.4 (0.2–0.9)	**0.026**
* LCE* > *35°*				
Acetabular retroversion^a^	0.7 (0.3–1.8)	0.451		
Female Sex	1.9 (1.2–3.1)	**0.008**		
Tönnis > 0	0.9 (0.5–1.6)	0.753		
Age > 40 years	0.6 (0.3–1.1)	0.080		
Severe cartilage damage	2.4 (1.4–3.9)	< **0.001**	3.6 (2.0–6.7)	< **0.001**

*MRA* magnetic resonance arthrography, *LCE* lateral center-edge angle

Bold values indicate statistical significance *p* < 0.05

^a^ Defined as retroversion index of > 30%, cross-over, and ischial spine sign positive

**Table 5:** Single-factor and multiple logistic regression analysis with corresponding odds ratios for the probability of femoral head decentration on radial images in indirect MRA

Parameter	Single-factor logistic regression		Stepwise multiple logistic regression	
	OR (95% CI)	*p*-value	OR (95% CI)	*p*-value
Hip dysplasia	6.3 (2.2–17.9)	< **0.001**	5.2 (1.7–16.0)	**0.004**
* LCE* < *25°*				
High femoral torsion	12.0 (1.2–121.3)	**0.035**	14.7 (1.2–175.7)	**0.034**
* >* *35°*				
Valgus deformity	1.4 (0.3–5.6)	0.658		
* Neck shaft angle* > *140°*				
Cam deformity	2.1 (0.8–5.8)	0.154		
* Alpha angle* > *55°*				
Acetabular overcoverage	0.3 (0.1–1.0)	**0.042**		
* LCE* > *35°*				
Acetabular retroversion^a^	0.5 (0.1–2.5)	0.407		
Female sex	0.9 (0.4–2.3)	0.883		
Tönnis > 0	0.9 (0.2–3.4)	0.839		
Age > 40 years	1.2 (0.4–3.3)	0.785		
Severe cartilage damage	4.0 (1.5–10.8)	**0.007**	4.3 (1.4–13.1)	**0.009**

*MRA* magnetic resonance arthrography, LCE lateral center-edge angle

Bold values indicate statistical significance *p* < 0.05

^a^ Defined as retroversion index of > 30%, cross-over and ischial spine sign positive

## Discussion

In the earliest description of FHD by Locher et al in 2002 the authors hypothesized that this sign was secondary to migration of the femoral head anteriorly into a large cartilage defect at the anterior acetabulum in patients with FAI [27]. More recently, FHD has been introduced as a possible sign of hip instability [5–7, 28–30]. Despite that, a concise description of the prevalence of FHD and its topographical distribution on different imaging planes is currently lacking. In our study presence of FHD was most frequently (both *p* < 0.001) detected on radial images followed by axial images for both direct MRA (30% versus 12%) and indirect MRA (22% versus 5%). More specifically, FHD was most commonly detected in the postero-inferior quadrant between 06:00 and 09:00 o’clock for both groups supporting the concept of migration of the femoral head towards the opposed antero-superior acetabulum (Fig. 3). Since the radial images allow for a circumferential perpendicular visualization of the acetabulum and proximal femur, it seems plausible that FHD, similar to cam deformities, is best visualized on this imaging plane [31].

In the literature prevalence of FHD on MRI varies considerably [6, 7, 29]. MacDonald and colleagues reported a prevalence of FHD in 3.6% (44/1227 hips) for non-contrast MRI and 3% (7/ 235) on direct MRA using a multiplanar protocol including radial images. By contrast, the prevalence of FHD on non-contrast MRI of the hip was 92% (46/50 hips) in a small cohort of patients with symptomatic hip dysplasia [29]. In our study compromising patients with FAI and hip dysplasia alike, we detected FHD in 30% (100/338 hips) on direct MRA and in 22% (22/109 hips) on indirect MRA. This is comparable to Zurmühle et al who performed direct MRA including radial images in patients with FAI and hip dysplasia and reported a prevalence of 29% (37/126 hips). To our surprise, the application of an intra-articular contrast agent and the subsequent joint distension was not associated with a higher prevalence of FHD. In fact, neither the prevalence of FHD (30% versus 22%, OR = 1.5 and *p* = 0.125) nor the mean decentration distance (1.4 mm versus 1.1 mm, *p* = 0.194) differed between direct or indirect MRA (Fig. 3).

One of the challenges when interpreting imaging findings in the setting of hip preservation surgery is the relatively high frequency of osseous deformities such as cam deformities and chondro-labral lesions in the asymptomatic population [32]. Interestingly, with a prevalence of 2% (1/43 hips) for FHD this was not the case in the asymptomatic participants supporting its potential usefulness to identify patients with hip pain.

The findings of our study support the hypothesis that FHD is associated with hip instability (Figs. 2 and 5). The majority of the 13 radiological parameters characterizing the proximal femur and acetabulum differed between hips with and without FHD in the direct and indirect MRA group (Table 3). A more detailed analysis of the direct MRA group showed that hips with FHD had bony deformities predisposing to hip instability [3, 33, 34]: These hips had reduced lateral coverage (LCE angle: 23 ± 9°), increased acetabular version (21 ± 7°), and increased femoral torsion (24 ± 15°). Our findings are confirmed by Macdonald et al [7] who reported similar mean values for the LCE angle 22.2 ± 7.8°, acetabular version of 19.2 ± 5.6° and femoral torsion of 22.2 ± 11.4° in 51 hips with FHD. Accordingly, on direct and indirect MRA, FHD was independently associated with instability-related deformities, such as hip dysplasia (OR = 6.1, *p* < 0.001 and OR = 5.2, *p* = 0.004) and high femoral torsion (OR = 3.0, *p* < 0.001 and OR = 14.7, *p* = 0.034) **(**Figs. 2 and 5).

In our study, severe cartilage damage was more frequently seen in hips with FHD. More specifically, FHD was associated with severe cartilage damage independent from the underlying osseous deformity for both direct MRA (OR = 3.6, *p* < 0.001) and indirect MRA (OR = 4.3, *p* < 0.009) (Tables 4 and 5). While previous studies did not specifically investigate the association between extensive cartilage damage and femoral head decentration [6, 29], MacDonald et al assessed chondral loss on MRI [7]. In their study with a relatively old population (mean 45.8 years), 82% (42 of 51 hips) had high-grade (grade 3 or 4) acetabular cartilage loss in the acetabular surface [7]. While Locher et al postulated a causal relationship of FHD being secondary to the femoral head migrating into an existing acetabular cartilage defect [27], our findings suggest a more complex relationship with FHD being independently associated with hip deformities related to instability and extensive intra-articular cartilage damage alike. In clinical practice, secondary migration of the femoral head due to extensive cartilage damage must be ruled out before FHD can be attributed to hip instability as FHD may be observed even in arthritic hips in the setting of acetabular overcoverage (Fig. 6).

This study has several limitations. First, during the study period, non-contrast hip MRI was not performed in our institution. Instead, we selected patients undergoing indirect MRA to assess whether the absence of joint distension affects the prevalence of FHD when being compared to the direct MRA group. This was not the case, instead, osseous hip deformities and severe cartilage damage were associated with FHD. However, we acknowledge the fact indirect MRA of the hip is not regularly performed nowadays due to potential systemic side effects. Therefore, our findings need to be confirmed on a non-contrast MRI of the hip. Second, we can not rule out that greater variations in intra-articular volumes affect the presence of FHD as injection volume generally includes 12–16 mL which is the generally recommended injection volume. Third, there is no clear definition of hip instability [35]. Consequently, defining a stable or unstable hip solely on the basis of radiographic parameters may be overly simplistic. Future studies will need to assess whether the presence of FHD can aid in surgical decision-making when surgeons contemplate whether or not to perform a periacetabular osteotomy in borderline dysplastic hips [36] or concomitant femoral derotational osteotomies to correct excessively high femoral torsion [37].

In summary, FHD is best seen on radial MRA images in the postero-inferior joint space, and its prevalence is not affected by the method of contrast agent application. While rare in asymptomatic participants, FHD is associated with osseous deformities predisposing to hip instability, as well as with extensive cartilage damage in symptomatic patients. Accordingly, in the absence of concomitant extensive cartilage defects, FHD may be interpreted as a sign of hip instability in hips with a dysplastic acetabulum and increased femoral torsion.

### Supplementary Information

ELECTRONIC SUPPLEMENTARY MATERIAL

## Data Availability

The datasets used and/or analyzed during the current study are available from the corresponding author upon reasonable request.

## Abbreviations

CI: Confidence interval
FAI: Femoroacetabular impingement
FHD: Femoral head decentration
LCE: Lateral center edge
MRA: Magnetic resonance arthrography
OR: Odds ratio
SD: Standard deviation
