# Supplementary material for: Femoral head decentration on hip MRI: comparison between imaging planes, methods of contrast administration, and hip deformities
Source: Insights Imaging. 2024 Aug 1;15:184. doi: 10.1186/s13244-024-01777-7 (PMC11294319; doi:10.1186/s13244-024-01777-7)
Supplement: Supplementary file 1 — ELECTRONIC SUPPLEMENTARY MATERIAL [file 13244_2024_1777_MOESM1_ESM.pdf]

# Femoral head decentration on hip MRI: Comparison between imaging planes, methods of contrast administration, and hip deformities

## ELECTRONIC SUPPLEMENTARY MATERIAL

**Supplementary table 1**. Sequence protocol of the direct- and indirect MRA groups and the asymptomatic control group undergoing non-contrast MRI of the hip

| Sequence | Study group | Region | Repetition Time (ms) | Echo Time (ms) | Matrix | FOV (mm) | Flip angle | Slice Thickness (mm) | Bandwidth (Hz/Px) |
|---|---|---|---|---|---|---|---|---|---|
| axial T1-w TSE | MRA | Knee | 300 | 9.6 | 256 x 154 | 350 | 150 | 3 | 250 |
| axial T1-w TSE | MRA | Hip | 500 | 14 | 512 x 512 | 160 | 150 | 3 | 160 |
| sagittal PD-w TSE | MRA | Hip | 3100 | 50 | 320 x 224 | 150 | 150 | 2 | 230 |
| radial PD-w TSE | MRA | Hip | 1500 | 18 | 448 x 314 | 160 | 143 | 4 | 223 |
| coronal PD-w TSE | MRA | Hip | 2960 | 22 | 384 x 269 | 150 | 150 | 3 | 210 |
| axial-oblique T2-w DESS | control | Hip | 14.57 | 4.97 | 320 x 320 | 192 | 25 | 1 | 223 |

MRA = Magnetic resonance arthrography, TSE = turbo spin echo; DESS = double-echo steady state,

**Supplementary table 2.** Interobserver reliability for assessing femoral head decentration with Cohen's kappa

| **MR imaging plane** | **Direct MR arthrography** (100 hips) | **Indirect MR arthrography** (100 hips) | **Control group** (43 hips) |
|---|---|---|---|
| Radial | 0.96 (0.90 – 1.00) | 0.97 (0.90 – 1.00) | 1.00 (0.90 – 1.00) |
| Axial | 1.00 (1.00 – 1.00) | 1.00 (1.00 – 1.00) | 1.00 (1.00 – 1.00) |
| Sagittal | 1.00 (1.00 – 1.00) | 1.00 (1.00 – 1.00) | 1.00 (1.00 – 1.00) |

Values are depicted as quadratic weighted Kappa values (95% Confidence interval).
